# Potential roles of non-lymphocytic cells in the pathogenesis of IgG4-related disease

**DOI:** 10.3389/fimmu.2022.940581

**Published:** 2022-07-28

**Authors:** Shaozhe Cai, Ziwei Hu, Yu Chen, Jixin Zhong, Lingli Dong

**Affiliations:** Department of Rheumatology and Immunology, Tongji Hospital, Tongji Medical College of Huazhong University of Science and Technology, Wuhan, China

**Keywords:** IgG4-related disease, non-lymphocyte, immunoinflammatory regulation, ectopic lymphoid follicles, atypical antigen presentation

## Abstract

Studies have confirmed the involvement of a variety of lymphocyte subsets, including type 2 helper T lymphocytes (Th2) and IgG4^+^ B lymphocytes, in the pathogenesis of IgG4-related disease (IgG4-RD). Those lymphocytes contribute to the major pathogenetic features of IgG4-RD. However, they are not the only cellular components in the immunoinflammatory environment of this mysterious disease entity. Recent studies have suggested that various non-lymphocytic components, including macrophages and fibroblasts, may also play an important role in the pathogenetic process of IgG4-RD in terms of contributing to the chronic and complex progress of the disease. Therefore, the potential role of non-lymphocyte in the pathogenesis of IgG4-RD is worth discussing.

## Background of IgG4-RD

IgG4-RD is a newly defined autoimmune disease of the century. Single or multiple organs/tissues may be involved simultaneously or successively in the disease progress, including salivary glands, lacrimal glands, pancreas, biliary tract, kidney, lung, retroperitoneum, and etc. The main clinical manifestations are tume-factive enlargement of the affected organs, often accompanied with increased serum IgG4 level (1). The typical pathological characteristics of IgG4-RD are dense lymphoplasmacytic infiltration, storiform fibrosis, and obliterative phlebitis (2). Increased eosinophil infiltration can also be observed. According to 2020 revised Comprehensive Diagnostic Criteria and 2019 American College of Rheumatology/European League Against Rheumatism (ACR/EULAR) classification criteria of IgG4-RD, fibrosis and lymphoplasmacytic infiltration are essential indices for the diagnosis of IgG4-RD (3, 4). The exact pathogenesis of IgG4-RD is still in the exploratory stage. Recent studies have suggested that multiple lymphocyte subsets including type 2 helper T cell (Th2), follicular helper T cell (Tfh), CD4^+^ cytotoxic T cell (CD4^+^ CTL), and B cells are involved. The interactions among these cells eventually mediate inflammation and fibrosis, contributing significantly to the pathogenesis of IgG4-RD (5–8).

Although, as an autoimmune disease, interactions between T and B cells contribute the majority of the pathogenesis in IgG4-RD (9), however, they are not the only cellular subsets involved in the real immuno-inflammatory response: residential parenchymal, mesenchymal cells, and other inflammatory cells infiltrated into the involved tissues, can respond to the stimuli derived from the local inflammatory microenvironment and further participate in the inflammatory response, which finally contribute to the histopathological features of IgG4-RD. Recent studies have also indicated that several non-lymphocytes, including macrophage and basophil, may exert important effects on the pathogenesis of IgG4-RD (10–12). Therefore, exploring the potential role of non-lymphocytes is crucial for in-depth understanding to the pathogenesis of IgG4-RD. This review will discuss this issue from the following three perspectives, based on the current researches in the field of IgG4-RD and other related conditions.

## Non-T/B lymphocyte-mediated fibrosis

**Figure 1:**
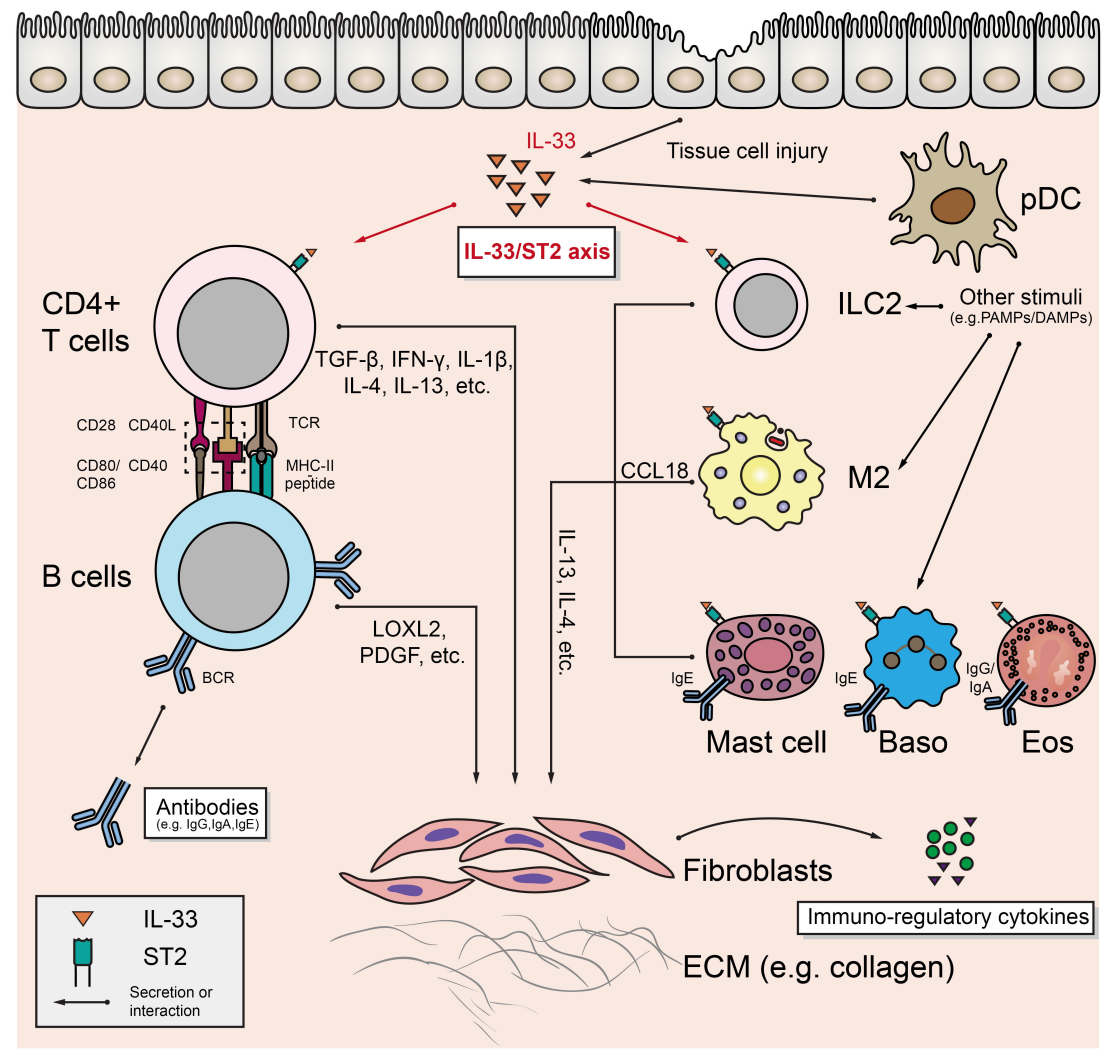
The underlying mechanism of fibrosis in IgG4-RD and the role of IL-33/ST2 axis.

As mentioned above, profibrotic factors (e.g., IL-1β, TGF-β, LOXL2, PDGF, etc.) production resulting from the interactions between T (primarily CD4^+^ CTL) and B cells is the core fibrogenic mechanism of IgG4-RD (13). However, a growing number of researches have revealed that innate immune cells may play important roles in this process (**Figure 1**).

### Alternatively activated macrophage

The alternatively activated macrophage (AAM), also known as M2 macrophage, is induced by typical type 2 cytokines (such as IL-4 and IL-13), and can be found in several pathophysiological conditions such as allergy, parasitic infection, and the maintenance of metabolic homeostasis in adipose tissue (14). Ishiguro N et al. found that TLR7 (mainly located on M2 in the IgG4-RD affected tissues) activation *in vitro* lead to enhanced interleukin 33 (IL-33) production by M2, which was also confirmed by the finding that human Toll-like receptor 7 (huTLR7)-transgenic C57BL/6 mice had severer tissue fibrosis after receiving the treatment of resiquimod (R848) (12). M2 may also aggravate fibrosis through CC-chemokine ligand 18 (CCL18). Akiyama M et al. found that serum CCL18 level was significantly positively correlated with IgG4-RD RI (15). Furukawa S et al. found that CCL18 was co-localized with massive infiltrated M2 in IgG4-RD affected tissues, and positively associated with the fibrosis scores (16). Honda F et al. confirmed that blocking CCL8 (analog of human CCL18) in LAT^Y136F^ knock-in mice (an animal model of IgG4-RD) alleviated the degree of fibrosis in affected salivary glands, and further *in vitro* assays revealed that CCL8 could directly stimulate collagen production in mouse fibroblasts (17). M2 can also induce plasma cell recruitment by secreting tumor necrosis factor ligand superfamily member 13(TNFSF13/APRIL) and further promote fibrogenesis (18).

### Type 2 innate lymphoid cells

Innate lymphoid cells (ILCs) develop from common lymphoid progenitors (CLPs) in bone marrow. Except for the absence of antigen-specific receptor, ILCs are similar to T cells in terms of transcriptional and functional features. According to the expression of key transcription factors in their development and the pattern of cytokine secretion, ILCs can be divided into three groups: ILC1 (expresses T-bet and produces IFN-γ), ILC2 (expresses GATA3 and produces IL-4, IL-5, and IL-13), and ILC3 (expresses RORγt, and produces IL-22 or IL-17) (19, 20). Zhang P et al. found that the circulating ILC2 level in IgG4-RD patients was significantly higher than that in healthy controls and was positively correlated with the number of circulating regulatory T cells (Tregs). In addition, the expression levels of CD154, PD-1, and CXCR5 on ILC2 were also positively correlated with the level of B cells in peripheral blood, serum IgG4, and IgE (21). Although they didn’t directly demonstrate the effect of IL-13 and IL-4 secreted by ILC2 on IgG4-RD fibrosis, multiple studies suggested that IL-13 and/or IL-4 could mediate tissue fibrosis either directly (by acting on fibroblasts) or indirectly (e.g., by acting on alternatively activated macrophage) (22–24).

### Mast cells, eosinophils, and basophils

Mast cells, basophils (Baso), and eosinophils (Eos) share many similarities in the expression of surface receptors and cytokines: they all express immunoglobulin Fc receptors (Eos expresses FcγRII/CD32 and FcαRI/CD89 for IgG and IgA; Baso and mast cells express high-affinity IgE Fc receptor FcϵRI) and secrete typical Th2-type immune response-related cytokines (such as IL-4 and IL-13) to accelerate the development of fibrosis after activation (25). Although understanding to the detailed mechanism of these cells in IgG4-RD is still limited, the pathological features of IgG4-RD suggest potential roles of these cells in the fibrosis of IgG4-RD: in recent studies, mild to moderate eosinophil infiltration was observed in the affected tissues, and increased peripheral blood eosinophil count and serum IgE level were detected in IgG4-RD patients (2, 26, 27), which suggests that Eos may tightly related to the pathogenesis of IgG4-RD. Increased local IgE may also activate Baso and mast cells in affected tissues, which can further produce profibrotic factors to enhance the fibrosis process in IgG4-RD.

### Potential roles of IL-33/ST2 axis in IgG4-RD fibrosis

An important feature of the aforementioned cellular components is that they all express suppression of tumorigenicity 2 (ST2, also known as IL-1RL1), which is the receptor of a IL-1 family member, interleukin 33 (IL-33) (28, 29). IL-33 is constitutively and highly expressed in endothelial cells, epithelial cells, and fibroblast-like cells, and can be induced to express in several CD45^+^ cell subsets (e.g., macrophages) under the inflammatory stimulation (28). IL-33 can act as an alarmin to indicate the tissue injury: when cells are damaged, full-length IL-33 is released and transformed to highly active form to exert its effects as a cytokine, after being cleaved by proteases secreted by local inflammatory cells (such as chymase derived from mast cells) (28–30). Li D et al. reported that IL-33 released by bleomycin-injured lung epithelial cells induced the release of IL-13 from ILC2, and then promoted the differentiation of macrophage to IL-33 secreting M2, which further lead to the secretion of IL-13 and TGF-β, and promoted the pulmonary fibrosis (22). Besides in ILC2 and M2, the expression of profibrotic factor IL-13 can also be up-regulated in Th2 cells, mast cells, Baso, and Eos under the treatment of IL-33 (22, 31–34). These researches suggest that IL-33/ST2 axis may be the core axis connecting the fibrosis mediated by Th2-type immune responses. Despite that the potential profibrotic role of IL-33/ST2 axis has been mentioned in some studies of IgG4-RD (12, 21, 35), its main target cells and its temporal influencing order on potential target cells are unknown. In addition, although IL-33 is mostly associated with Th2-type immune responses in current studies, non-Th2-type cells, including Th1 and Treg cells, can also express ST2 and respond to IL-33 stimulation (36, 37). Therefore, the specific role of IL-33/ST2 axis in the pathogenesis of IgG4-RD still remains to be explored.

Beside promoting fibrosis, the above-mentioned immune cells may also influence the IgG4-RD immune response by other means such as secreting a variety of immunoregulatory agents: for example, IL-33 can enhance the activation of Th2 cells (32); Baso and monocytes can increase the production of IgG4 after the activation of some specific pattern recognition receptors (10, 38). Moreover, fibroblasts acting as collagen producing effector cells in this process can also exert other effects on the immunoinflammatory process of IgG4-RD (see below).

## Non-profibrotic effects of fibroblasts

**Figure 2:**
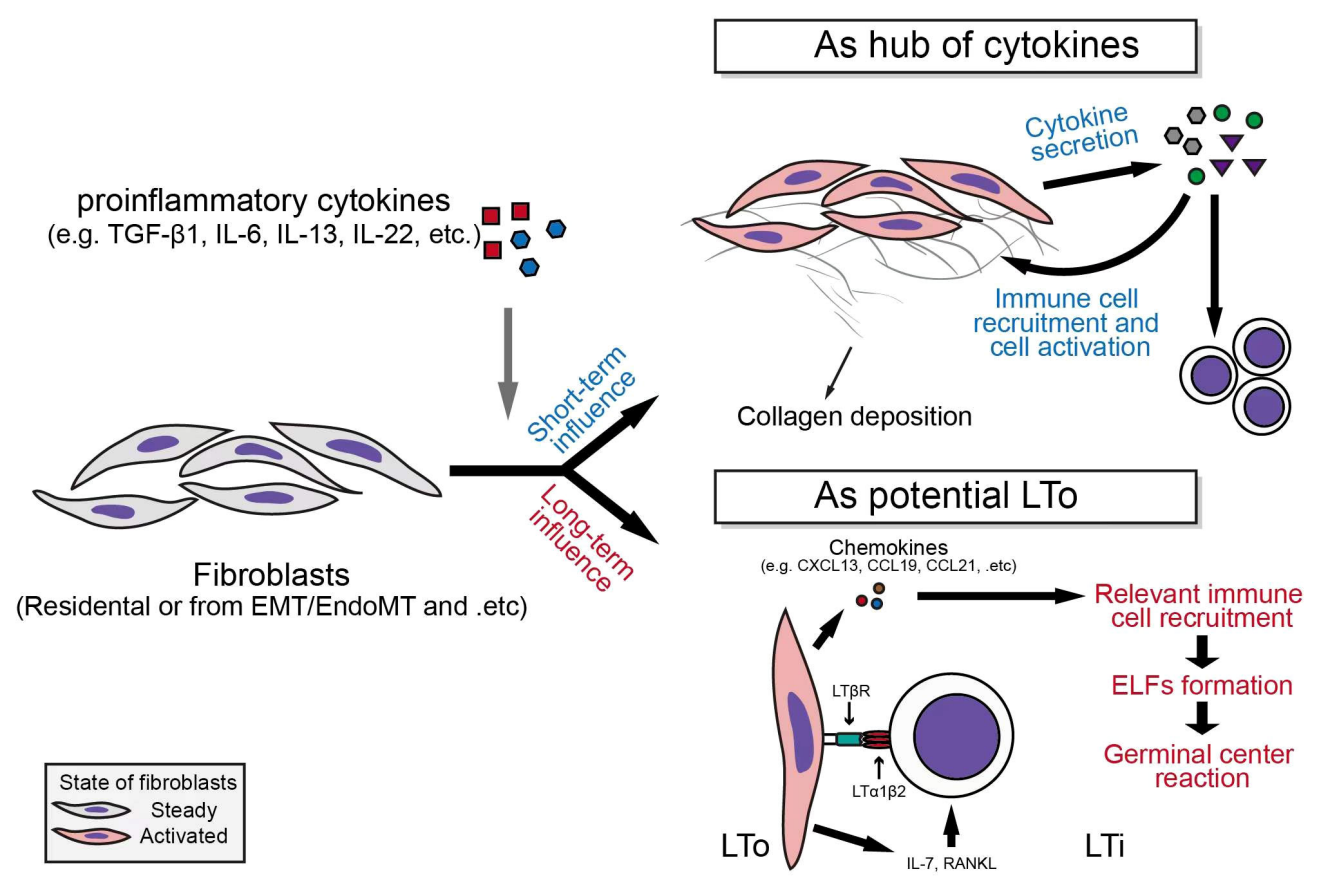
Fibroblasts serve as secretion centers of inflammatory factors and participate in the formation of ectopic lymphoid follicles.

Fibroblasts are non-hematopoietic, non-endothelial, non-parenchymal, non-epithelial and non-mesothelial cells with mesenchymal origin. However, fibroblasts can be derived from mesothelial-, epithelial-, or endothelial-to-mesenchymal transition, and may be transformed from hematopoietic cells (39). As illustrated above, fibrosis is one of the core pathological features of IgG4-RD. The transition of fibroblasts into ECM-producing myofibroblasts is a common feature of fibrosis-related diseases (40). However, in a dynamic and complex inflammatory response, activated fibroblasts can not only functionate as a “collagen maker”, but also participate in the regulation of immune processes (**Figure 2**).

### Fibroblasts can serve as a hub of cytokines

Fibroblasts express a variety of cytokine receptors (e.g., CCR7, CXCR2, IL-4R, IL-6R, IFN-γR, etc.) and pattern recognition receptors, which indicate their ability to receive inflammatory signal derived from different pathological environments, and also suggests their strong potential to make response to the stimulation of these receptors (41–43). For example, in malignant tumors, cancer-associated fibroblasts (CAFs) serve as the main source of inflammatory factors including CXCL12, CXCL1, and IL-6, and participate in the regulation of immune responses in various malignant tumors (e.g., pancreatic ductal adenocarcinoma) (44). In classic fibrotic diseases, such as idiopathic pulmonary fibrosis (IPF), fibroblasts can up-regulate the expressions of IL-6, IL-8, and CCL2, under the stimulation of IL-1β (45); Furthermore, the expression of CCL5 was also up-regulated in response to CCL21 stimulation in primary lung fibroblasts derived from IPF patients (46). In Dupuytren’s disease, tumor necrosis factor α (TNFα) stimulates myofibroblasts to secrete IL-33, which in turn increase the secretion of TNFα from other immune cells, and finally forms a vicious circle (47).

The important roles of fibroblasts in autoimmune conditions have also been demonstrated in human specimens and animal models. In serum transfer induced arthritis (STIA) K/BxN mouse model, the secretion of cytokines by THY1^+^ fibroblasts of hip joint, including CXCL12, CCL2, CCL5 and IL-6, is significantly enhanced under the stimulation of TNFα (48). TNFα, IL-1β, and hypoxia also up-regulate the expression of cytokines, including IL-6, CXCL8 (namely, IL-8), CCL2, CXCL11, CXCL12, and VEGF, as well as the expression of cytokine receptors such as TNFRSF9 in synovial fibroblasts derived from rheumatoid arthritis (RA) patients (49, 50). In systemic sclerosis (SSc), IL-22 synergistically works with TNFα to induce the expression of IL-8 and CCL2 in skin fibroblasts, while the simultaneous stimulation of TGF-β1 and IL-17A can lead to an up-regulation of IL-6 expression in fibroblasts from affected skin by nearly a hundredfold (51, 52).

In involved tissues of IgG4-related retroperitoneal fibrosis (IgG4-RPF), the co-localization of IL-6, BAFF, IL-7, IL-12 p70 and IL-23 with αSMA is observed by Zongfei J et al, and the expression levels of both IL-6 and IL-6R are higher than those in the control tissues, suggesting the pro-inflammatory properties of fibroblasts in affected tissues of IgG4-RD patients (53). Elevated secretion of BAFF, IL-7, IL-12 p70, and IL-23 in the aortic adventitial fibroblasts (AAFs) after the activation of IL-6/IL-6R axis can also be seen (53). It is worth mentioning that the increased production of IL-7, IL-12, IL-23, and other cytokines by IgG4-RD fibroblasts can not only play an immediate role in regulating the immunoinflammatory processes *via* affecting the functional status of infiltrated immune cells, but also can exert a long-term impact on the progress of autoimmune diseases by participating in the formation of ectopic lymphoid follicles (ELFs) (54).

### Fibroblasts may participate in the formation of ectopic lymphoid follicles (ELFs)

Ectopic lymphoid follicles, also known as tertiary lymphoid structures (TLSs), which is induced by inflammatory insults, and can contribute to the “propagation” of antigenic specific responses in local tissues (55). The developed ELF is a complex collection of white blood cells and specific mesenchymal cells, which is functionally similar to the secondary lymphoid organs (SLOs) including spleen and lymph nodes and provides a site for germinal center reaction (GC reactions) (54, 55).

The interaction between lymphoid tissue inducer (LTi) and lymphoid tissue organizer (LTo) is required for the generation of lymphoid follicles. LTis are developed from IL-7Rα^+^ lymphoid progenitors, and this is a process that requires the expression of retinoic acid receptor-related orphan receptor-γt (RORγt) (56). LTi cells express lymphotoxin α1β2 (LTα1β2) under the stimulation of IL-7 and RANKL (55, 56). *Via* binding to LTβ receptor (LTβR) expressed on LTo cells, LTα1β2 induces the release of chemokines (e.g., CXCL13, CCL19, and CCL21), as well as the expression of adhesion molecules, which further recruits immune cells, and promotes the formation of lymphoid structures (55, 57). In actual pathological environment, a variety of cells, including Th17 and Tγδ17, are involved in the formation of ELF (55). In chronic inflammation, a key step in the formation process of ELF is the transition of resident stromal cells (e.g., fibroblasts) to LTo-like phenotype after receiving the stimulation of inflammatory stimuli: for example, in synovial tissues of RA patients, the enhancement of IL-6 signal can lead to increased IL-7 production in synovial fibroblasts, which can induce LTi recruitment into the affected tissues and promote ELF formation (57–60).

TLS assists to eliminate pathogenic microorganisms when it is produced in local site of infections. However, in some autoimmune settings (e.g., Sjogren’s syndrome, systemic lupus erythematosus, etc.), the formation of TLS can aggravate the progress of autoimmune diseases through somatic hypermutation (SHM), isotype switching, and affinity maturation (56, 61). It is worth mentioning that Nayar S et al. found that during TLS formation in salivary glands in patients with Primary Sjogren’s Syndrome (pSS), the differentiation of a group of resident podoplanin (PDPN) positive mesenchymal cells into the “immunofibroblasts” that could support the early TLS formation happens before the infiltration of lymphocytes into the salivary glands, which is mainly regulated by IL-13 derived from resident ILCs and stromal cells (62). After lymphocyte infiltration, those immunofibroblasts were further phenotypically stabilized and proliferated under the influence of IL-22 and lymphotoxin (62). These studies show that innate immune cells may have already been primed for the potential chronicity of adaptive immune response before it occurs, and provide important hints for the potential mechanism of ELF formation in autoimmune diseases with mucosa containing organs’ involvement.

ELF probably plays an important role in the pathogenesis of IgG4-RD (63): there is marked fibroblast activation in IgG4-RD involved tissues, and Zongfei J et al. observed that fibroblasts in affected tissues could express IL-7 (an ELF formation promoting cytokine) after the activation of IL-6/IL-6R axis (53); Chen Y et al. found an abnormal expansion of Tfh cells in IgG4-RD patient tissues (5); In addition, infiltration of a large amount of B cells into the affected tissues, and the diversity of B cell clones suggest the possible existence of ectopic germinal center reactions in IgG4-RD (64, 65); Zhang P et al. observed the infiltration of ILC in the affected tissues of IgG4-RD patients, and the positive correlation of ILC2 with the disease severity of IgG4-RD (21). All these aforementioned components can be involved in the formation of ELF and participate in the subsequent immunoinflammatory reactions. Considering the predisposition of exo-/endocrine gland involvement (mucosa containing organs), marked fibroblast activation (potential LTo), and dense infiltration of immune cells (potential LTi) in IgG4-RD, the possible role of fibroblasts in the formation of ELF in the pathogenesis of IgG4-RD is worthy of further investigation. Besides, fibroblasts can express major histocompatibility complex class II (MHC-II) molecules under the influence of inflammatory stimuli, which leads them become the target cells of CD4^+^ CTLs resulting in apoptosis and the subsequent biological processes (see below) (66).

## Atypical antigen presentation

Antigen presentation is an important immunological process that assists T cells in antigen recognition and activation. Antigen-presenting cells (APCs) can be grouped into professional APCs and non-professional APCs based on the expression of different MHC molecules. Professional APCs include dendritic cells (DCs), macrophages, and B cells (67). Most nucleated cells can present antigen to CD8^+^ T cells *via* MHC-I molecules, while professional APCs present antigen peptides to CD4^+^ T lymphocytes through their constitutively expressed MHC-II molecules (68). With the help of CD4/CD8, and other surface molecules such as CD45, the binding of T cell receptor (TCR) to antigen peptide-MHC complex lead to the phosphorylation of immunoreceptor tyrosine-based activation motif (ITAM) on the cytoplasmic tail of CD3, and initiates the downstream TCR signaling (69).

However, in some pathological conditions, the expression of MHC-II molecules can be induced on some non-professional APCs by inflammatory stimuli (namely, ectopic expression). Winau F et al. found that IFN-γ could up-regulate the expression of MHC-II in hepatic stellate cells (HSC) (70). Kato et al. found that the MHC-II expression on synovial fibroblasts was increased when co-cultured with Th1 cells (71). Even human T cells can express MHC-II molecules and present autoantigenic peptides after activation of TCR signaling (72). In addition to the aforementioned cells, cells including Eos, Baso, and epithelial cells may express MHC-II molecules under the stimulation of different inflammatory stimuli, which is well reviewed by Kambayashi T and Laufer TM (73).

**Figure 3:**
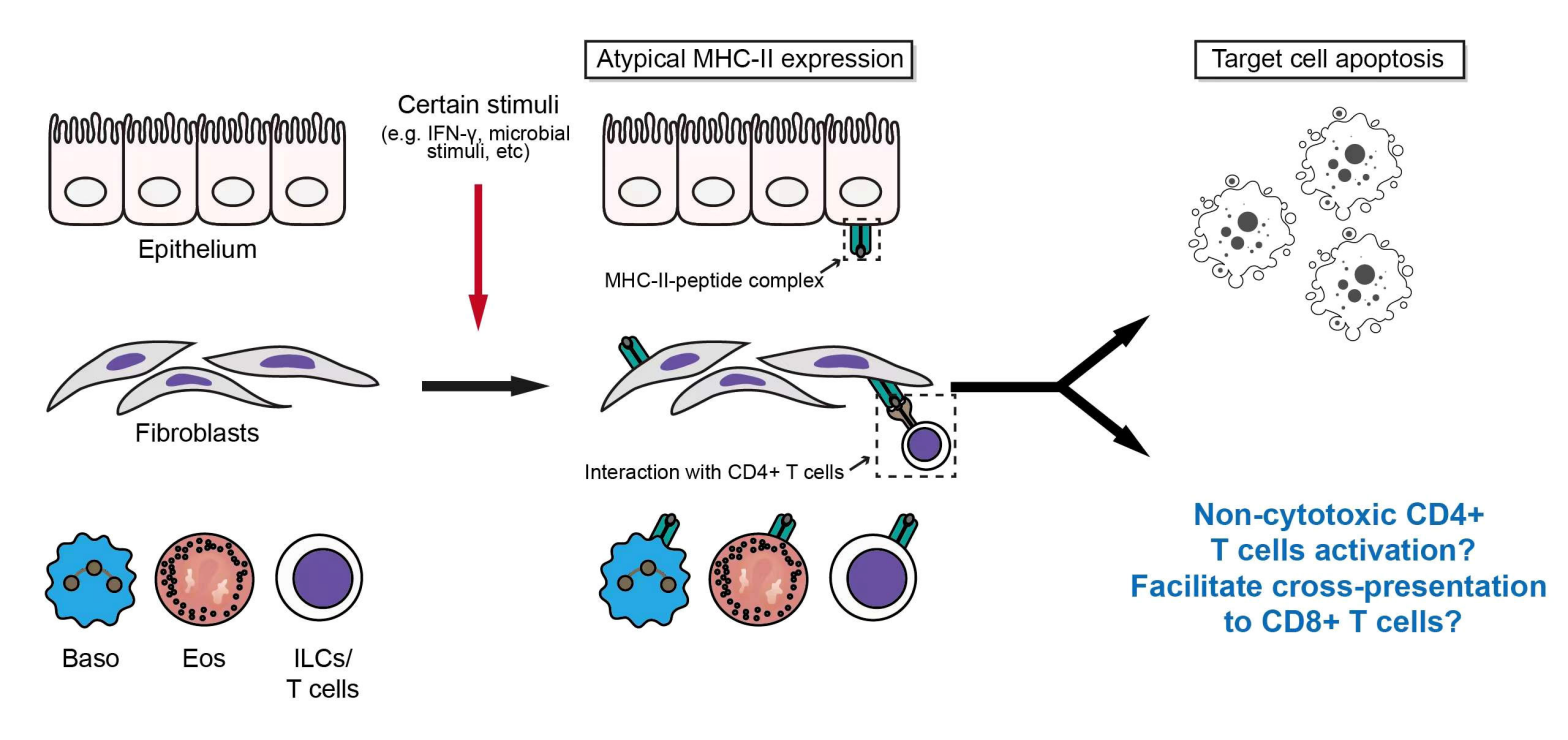
Induction of atypical antigen presentation and its potential effect on IgG4-RD.

In IgG4-RD, Perugino CA et al. found that CD4^+^ CTL mediated the apoptosis of a variety of cells in affected tissues, including T cells, B cells, acinar cells, ductal cells, and endothelial cells, and the expression level of HLA-DR in these apoptotic cells was significantly increased (66). Interestingly, those apoptotic cells are mainly non-endothelial, non-immune cells with mesenchymal origin (66). However, the regulatory mechanisms and specific roles of atypical MHC-II expression in these cells remain unknown in IgG4-RD. Considering the existence of the previously mentioned inducing factors for the atypical expression of MHC-II molecules (e.g., infiltration of immune cell subsets, and immunoinflammatory microenvironment) (7, 10, 21, 74) and the potential influence of environmental factors on IgG4-RD (75), atypical antigen presentations *via* MHC-II molecules may be the factor to promote or even initiate the development of IgG4-RD. In addition, inflammatory microenvironment could also up-regulate the expression of MHC-I molecules, while the dying cells induced by CD4^+^CTL may contribute to cross-presentation, which may be an important basis for the potential pathogenic role of CD8^+^ T cells in IgG4-RD (66, 76, 77) (**Figure 3**).

## Summary and prospect

Non-lymphocyte can exert important effects on the immune-inflammatory regulations in many pathological conditions, such as RA, SLE, SSc, and etc. (78–80). Current studies on IgG4-RD and basic immunological evidence suggest that non-lymphocyte may play roles in the initiation of IgG4-RD disease (such as atypical antigen presentation), disease severity (such as mediating fibrosis and participating in the regulation of immune inflammation), as well as chronic and complex progress (such as the formation of ectopic lymphoid follicles). Although more and more evidence suggest the potential role of non-lymphocyte in the pathogenesis of IgG4-RD, the research on IgG4-RD is still very limited at present in this area, mainly because IgG4-RD is a newly defined disease entity in this century, and there is no particularly suitable animal model for IgG4-RD investigation. Animal models that have been published so far include the patient serum injection model proposed by Shiokawa et al. (injecting serum IgG of IgG4-RD patients into male Balb/C neonatal mice), LAT^Y136F^ knock-in mouse model identified by Yamada et al., and Human TLR7 transgenic mouse model proposed by Ishiguro N et al. (12, 81, 82). These models may assist in exploring the potential roles of non-lymphocyte in IgG4-RD *in vivo*. Considering the lack of appropriate animal models, the application of high-throughput techniques coupled with well-designed experiments may help further mechanistically elucidating the functional status of these cells and their association with IgG4-RD: *via* single-cell transcriptome sequencing (scRNA-Seq), Valenzi E et al. identified the heterogeneity of fibroblasts in patients with systemic sclerosis associated interstitial lung disease (SSC-ILD) revealing that myofibroblast differentiation and proliferation were the key pathological mechanisms promoting fibrosis in patients with SSC-ILD (83); Der E et al. found that the signal signatures of type I interferon and the fibrosis-related signaling in renal tubular cells and keratinocyte of lupus nephritis patients were differentiated from that in healthy controls (84). It is worth mentioning that detailed investigation into the pathological roles of non-lymphocytes may help provide helpful therapeutic insights, not only for IgG4-RD, but also for many other autoimmune conditions: different diseases may share some pathogenic cytokines, which have the potential to be the therapeutic targets. For example, besides in IgG4-RD, IL-33/ST2 axis has been identified to play roles in several fibrotic process related diseases (e.g., pSS), and antibodies targeting have been utilized in the clinical trials of several diseases, including atopic dermatitis, and asthma (80, 85); type I interferon can also exert pathological effects and be the therapeutic target in several autoimmune conditions (e.g. SLE) (86). In consideration of the fact that the components of non-lymphocytes may affect the pathogenesis of IgG4-RD at multiple levels, further mechanistic researches and large-scale clinical association analyses focusing on the specific roles of different non-lymphocytes in the initiation and progress of IgG4-RD will help indicate the optimal usage of target therapy, which in turn, on this basis, will deepen our understanding to the potential pathogenic roles of non-lymphocyte and related inflammatory factors in IgG4-RD.

## Author contributions

SC and ZH performed literature search and drafted the manuscript. YC, JZ, and LD revised the manuscript. All author designed and reviewed the manuscript. All authors contributed to the article and approved the submitted version.

## Funding

This work was supported by grants from the National Natural Science Foundation of China (81974254 and 81901651), and Tongji Hospital Clinical Research Flagship Program (No. 2019CR206).

## Conflict of interest

The authors declare that the research was conducted in the absence of any commercial or financial relationships that could be construed as a potential conflict of interest.

## Publisher’s note

All claims expressed in this article are solely those of the authors and do not necessarily represent those of their affiliated organizations, or those of the publisher, the editors and the reviewers. Any product that may be evaluated in this article, or claim that may be made by its manufacturer, is not guaranteed or endorsed by the publisher.
